# Perceived quality of care for severe acute malnutrition management among caregivers of under-five children with severe acute malnutrition in Addis Ababa, Ethiopia, 2022: a mixed-method study

**DOI:** 10.3389/fpubh.2023.1089323

**Published:** 2023-04-26

**Authors:** Bulcha Guye Adema, Niguse Tadele Atnafu, Feven Mulugeta Ashagre

**Affiliations:** ^1^Department of Pediatrics and Child Health Nursing, School of Nursing, College of Health Sciences, Wolaita Sodo University, Sodo, Ethiopia; ^2^Departments of Nursing, School of Nursing and Midwifery, College of Health Sciences, Addis Ababa University, Addis Ababa, Ethiopia

**Keywords:** quality of care, severe acute malnutrition (SAM), Addis Ababa, Ethiopia, moderate malnutrition

## Abstract

**Introduction:**

All the factors at the institutional, provider, and client levels have an impact on the quality of care. In low- and middle-income countries, poor quality of severe acute malnutrition (SAM) management at health institutions is a major contributor to child morbidity and mortality. This study aimed to determine the perceived quality of care for SAM management among caregivers of under-five children.

**Methods:**

This study was conducted in public health facilities that provide inpatient SAM management in Addis Ababa, Ethiopia. An institution-based convergent mixed-method study design was implemented. Quantitative data were analyzed by using a logistic regression model, while thematic analysis was used to analyze the qualitative data.

**Results:**

A total of 181 caregivers and 15 healthcare providers were recruited. The overall perceived quality of care for SAM management was 55.80% (CI: 48.5–63.10). Urban residence (AOR = 0.32, 95% CI: 0.16–0.66), college and above level education (AOR = 4.42, 95% CI: 1.41–13.86), working as a government employee (AOR = 2.72, 95% CI: 1.05–7.05), readmitted to the hospital (AOR = 0.47, 95% CI: 0.23–0.94), and length of hospital stays >7 days (AOR = 2.1, 95% CI: 1.01–4.27) were found to be significantly associated factors with perceived low-quality care for SAM management. Additionally, lack of support and attention from higher levels of management, and lack of supplements, separate units, and laboratory facilities were among the factors that impede the provision of quality care.

**Discussion:**

Perceived quality of SAM management services was low against the national goal of quality improvement to meet the expectations of both internal and external clients. Rural residents, those with more educational qualifications, government employees, newly admitted patients, and patients who stayed longer in hospitals were the most unsatisfied groups. Improving support and logistic supply to health facilities, providing client-centered care, and responding to caregivers' demands may help to improve quality and satisfaction.

## Introduction

The Institute of Medicine (1) defines healthcare quality as the extent to which health services for individuals and populations increase the likelihood of desired health outcomes while remaining consistent with current professional knowledge (2). As a result of advancements in technology and medical sciences in today's world, healthcare organizations are in high demand to provide quality healthcare services (3).

Hypoglycemia, infection, anemia, dehydration, hypothermia, electrolyte imbalance, HIV and tuberculosis infection, age, and sex were among the factors linked to a high death rate in children with SAM, and these complications result in poor-quality SAM care. We must focus on providing high-quality treatment for SAM management in order to improve pediatric outcomes (4, 5).

Quality in healthcare is the result of patient and healthcare practitioner collaboration in a supportive environment. Healthcare service quality is influenced by the personal aspects of the clinician and the patient, as well as factors relevant to the healthcare organization, healthcare system, and the larger environment (2, 6).

Service quality is seen as a critical aspect of an organization's success and survival since it assures user loyalty and preserves its competitive edge. Customers who obtain high-quality healthcare services are more likely to trust and return to the healthcare provider. The quality of healthcare services also plays an important role in the successful implementation of universal health coverage (7, 8).

Quality treatment for the management of SAM in children has received less attention, and factors associated with program quality have not been studied in-depth (9, 10). Acute malnutrition was previously perceived as a humanitarian emergency rather than a development and public health concern, despite the long-term economic and social costs associated with the condition. However, more attention is being paid to the problem, and it is now recognized as a public health and development priority (11).

Treatment coverage must be considerably improved by removing barriers to access to satisfy global SAM demands. Lack of community awareness of malnutrition programs, high opportunity costs, and distance to treatment options are all common obstacles (12).

Countries' capacity to provide high-quality care for SAM management is determined by the availability of infrastructure and well-trained staff in public hospitals dedicated to the treatment of malnourished children (13). Poor quality of care (QOC) has been implicated as a major risk factor for high mortality across conditions, especially in low- and middle-income countries (14).

Case fatality rates (CFRs) in hospitals treating SAM remain at 20–30% in most underdeveloped countries, and only a small percentage of individuals who need treatment receive it (15). The Delphi process indicated that adherence to standardized protocols for the treatment of SAM and MAM should have a marked positive impact on mortality and recovery rates; yet, the true consensus was not achieved (16).

Approximately 17 million children worldwide suffer from SAM (17). Globally, in 2020, 149 million under-five children were estimated to be stunted (too short for age), 45 million were estimated to be wasted (too thin for height), and 38.9 million were overweight or obese. Approximately 45% of deaths among children under 5 years is linked to under-nutrition (22). Low- and middle-income countries bear the greatest burden of malnutrition, especially those in Sub-Saharan Africa and Asia (18).

According to the 2019 Ethiopian Demographic and Health Survey (EDHS) report, among children under 5 years in Ethiopia, 7% were wasted, 37% were stunted, and 21% were underweight. Regional variations exist, with the highest percentages of children who are wasted in Somali (21%), Afar (14%), and Gambela (13%) and the lowest percentages of wasted children in Addis Ababa (2%) and Harari (4%) (19).

In 2016, LMICs experienced 15.6 million extra fatalities due to 61 conditions. After removing deaths that might have been prevented with public health measures, there were 8.6 million extra deaths that could have been prevented with healthcare, of which 5.0 million were due to poor-quality care and 3.6 million were due to non-use of healthcare.

Poor quality of pediatric healthcare services at health facilities is a major contributing factor to child morbidity and mortality in low- and middle-income countries, including Ethiopia, and it brings parental dissatisfaction. Child mortality may be influenced by the poor quality of treatment offered in health institutions. Poor-quality care can also lead to other negative consequences, such as a lack of trust in the healthcare system (6, 20, 21).

Universal health coverage has been proposed as a strategy for improving population health. However, the success of this strategy is also dependent on the provision of high-quality healthcare (22). Poor-quality care, estimated to cost trillions of dollars each year, is holding back progress on improving health in countries at all income levels. Without quality health services, universal health coverage will remain an empty promise (23).

It was expected that adherence to the guideline and steps by healthcare providers would reduce mortality to <10% (24). The effects of malnutrition on health, education, production, risk for cardiovascular disease, accidents to newborns, and communicable diseases are based on the relative risk faced by those who are malnourished in their early years of life (25, 26).

Even though integrated pediatrics healthcare service initiatives have been developed in most Ethiopian hospitals, the delivery of quality remains a concern SAM. The quality of care provided to children with SAM is extremely beneficial in lowering mortality rates among patients with SAM. To the best of my knowledge, Ethiopia has very little evidence on the matter.

Improving the quality of care is one of the curial steps in healthcare system special for SAM management. Universal health coverage has been proposed as an approach to improving population health. However, the effectiveness of this technique is partly contingent on the availability of high-quality healthcare.

This study will contribute data on the perceived quality of care for SAM management in the study area, as well as increase the quality service for the patients received from healthcare providers and assess healthcare professionals on the perceived quality care SAM management. The findings of this study will assist healthcare administrators and other stakeholders in emphasizing the problem of SAM, identifying gaps in SAM management, and employing high-quality SAM to evaluate the efficacy of inpatient care as a baseline for future interventional methods. It also acts as a starting point for future studies.

## Methods and materials

### Study area and period

The study was conducted from 15 February to 20 May 2022 in Addis Ababa, which is the capital city of Ethiopia, and the seat for the African Union & European Economic Commission, with a population of 3,384,569 in an area of 540 square kilometers (27). There are 93 public health facilities in Addis Ababa, and this study included all four public hospitals which provide in-patient services for SAM management, namely, Yekatit 12 medical college Hospital (Y12MCH), Menelik II General Referral Hospital (MIIGRH), Zewditu Memorial General Hospital (ZMGH), and Tirunesh Beijing General Hospital (TBGH).

### Study design

A facility-based convergent mixed-method design was employed. Both quantitative and qualitative data were collected and analyzed, and then the analysis of quantitative and qualitative data was compared. The intent of integration of quantitative and qualitative data collection methods was chosen to enhance the value of our methodology as it was noted by previous researchers (28, 29).

### Study population

For the quantitative survey, caregivers of under-five children with SAM admitted to the hospital during the study period who were taking care of the child as a primary caregiver were included and those who were unable to communicate in Amharic were excluded from the study. For the qualitative survey, purposively selected healthcare providers who have at least 6 months of experience in the management of SAM at the pediatric ward of selected facilities were included.

### Sample size determination and sampling procedure

For the quantitative survey, the required sample size was determined by using the single population proportion formula with the following assumptions: proportion (p) = 57.6% (proportion of caregivers perceived high quality of pediatric healthcare services) [5}, Z *a/2* at 95% confidence level = 1.96, and margin of error = 5%. Based on the above assumptions and by adding 10% of the calculated sample size for the non-respondent rate, the total sample size was estimated to be 413 caregivers. For the qualitative survey, the sample size was determined by data saturation point as per sample size guidelines in qualitative research (30).

**Figure 1:**
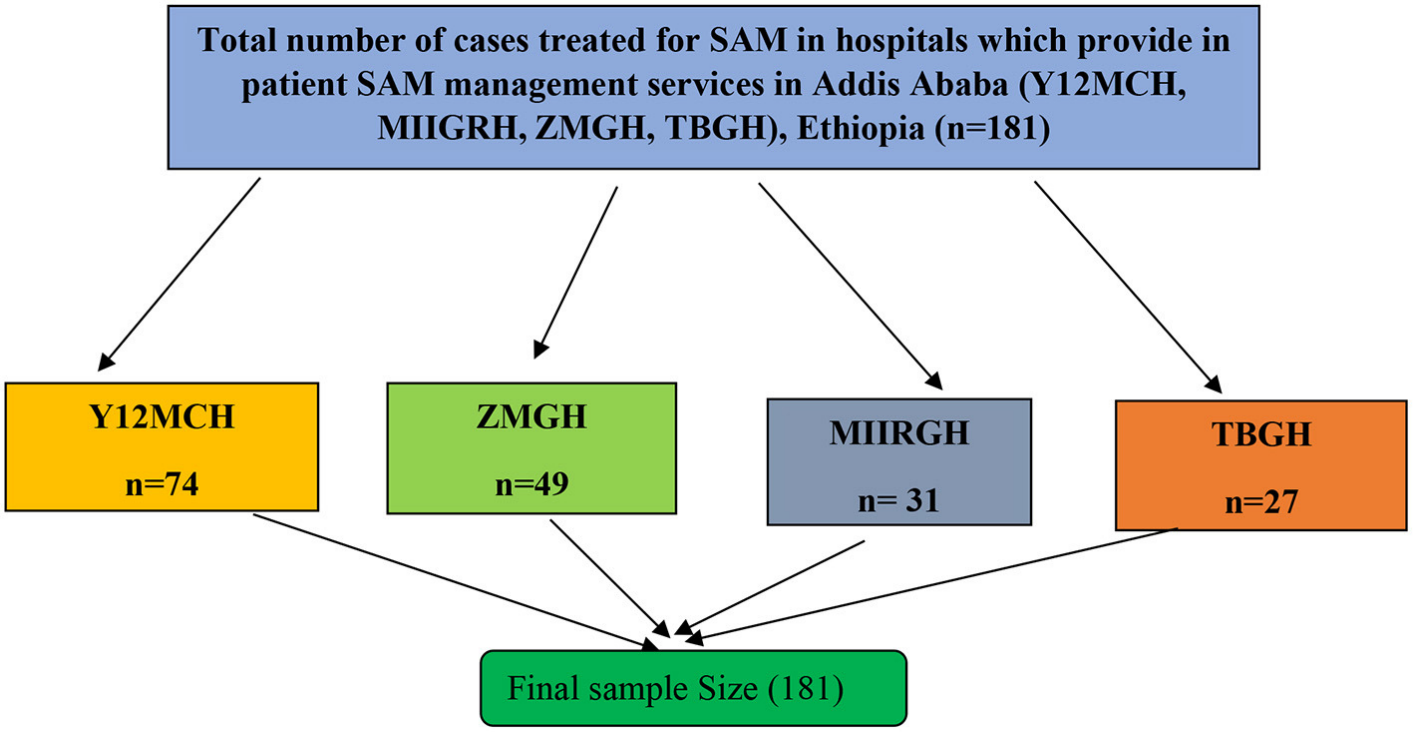
Schematic presentation of sampling procedure for a study on quality of care for SAM management among caregivers of under-five children in selected public hospital in Addis Ababa, Ethiopia, 2022. Y12MCH, Yekatit 12 Hospital Medical College; ZMGH, Zewditu Memorial General Hospital; MIIGRH, Menelik the Second General Referral Hospital; TBGH, Tirunesh Beijing General Hospital.

A stratified sampling technique was used for the quantitative study. Caregivers were stratified based on the hospital they were admitted to, and the caregivers of all under-five children who were managed for SAM were interviewed consecutively from each stratum (Figure 1). For the qualitative survey, the purposive sampling method was used to select healthcare providers who could provide rich information on the research questions based on their experience and position. Before inclusion, screening interviews were conducted to assure the eligibility of respondents for the in-depth interview, and interviews were continued until reaching data saturation, and no new data were obtained from respondents.

### Study variables and data collection tools and procedures

The outcome (dependent) variable for this study was the perceived quality of care for SAM management. The independent variables were socio-demographic characteristics (residence, sex, age, educational status, occupation, and marital status), length of hospital stay, and types of admission.

For the qualitative survey, the English version questionnaire that comprised five dimensions was adapted from the validated tool of the HEALTHQUAL model (6, 31). The questionnaire was first translated into Amharic, and then, the reverse translation was done into English by language experts to check for its accuracy. Additionally, it was pre-tested in Black Lion Comprehensive Specialized Hospital on 5% of the sample to improve the questionnaire's simplicity of understanding and flow, and amendments were made to its flow. Data were collected using a face-to-face interview with four BSc nurses from other facilities who have data collection experience by using electronic data collecting application [Open Data Kit (ODK) version 1.0] with the server of the Kobo Toolbox. To verify internal consistency, a scale reliability analysis was undertaken by computing Cronbach's alpha values for the overall and each subscale. The subscales' Cronbach's alpha values were above 0.70, and the total scale was 0.71, indicating that there was great internal consistency. Inter-item correlation coefficients among items were lower than 0.60, reflecting that each item independently measures the unique characteristic of service quality.

For the qualitative data collection, face-to-face in-depth interviews were conducted using the semi-structured open-ended interview guide. The interview guide featured a set of questions with probes to help drive the interview in a conversational manner and in a specific direction. It also included questions on socio-demographic characteristics, the SAM management process, quality of care for SAM, and factors affecting the quality of care in the management of SAM. A research assistant who had qualitative data collection experience conducted the interviews in a separate place to minimize interruptions in Amharic, and the interviews were audio-recorded for ease of transcription.

## Trustworthiness

### Credibility

To preserve data source triangulation, in-depth interviews were conducted in a confidential and comfortable setting among healthcare providers in a selected hospital. Member check was used after the formation of primary codes. The audio tape was transcribed in Amharic word to word before being translated into English. On the same day, data transcription and analysis were completed.

### Dependability

Reviewing the audio recording and writing notes helped to confirm the manual transcription. The procedures of data collection, processing, and study findings were assessed by people who did not participate in the data analysis.

### Transferability

The investigator provided many details to explain the entire research procedure, from data collection to the final report.

### Conformability

To eliminate bias throughout data collection, coding, and analysis, the researcher reflected on and examined prior personal expectations and experiences to achieve conformability. The participants' own words were used instead of the researchers' opinions and biases.

### Data processing and analysis

*Quantitative data were collected using ODK version 1.0, exported to* Microsoft Excel and *Microsoft* W*ord, and then transported to STATA version 16 for analysis*. Descriptive statistics such as frequency, mean with standard deviation for normally distributed data, and median with interquartile range for non-normally distributed data were computed for all variables related to the objective of the study. There was no multicollinearity when the collinearity diagnostic test was performed on the independent variables (a variance-inflation factor (VIF) of > 5 and tolerance value of <0.10 was used as the cut point to test multicollinearity). Cross-tabulation was done to see the distribution of each independent variable by the independent variable.

Simple and multiple binary logistic regression analyses were computed to assess associations between dependent and independent variables. Variables that had a *P*-value of <0.25 in the bivariable logistic regression analysis were fitted for multivariable analysis. In bivariable logistic regression analysis, variables with a *P*-value of <0.25 were fitted for multivariable analysis. The chi-square value for the Hosmer–Lemeshow test was 5.04 with a significance level of 0.7532 and was not statistically significant, indicating that the model was a perfect fit. The strength of the associations was interpreted using an adjusted odds ratio (AOR) with a 95% confidence interval (CI). Finally, the findings of the study are displayed by using texts, tables, and figures.

For the qualitative survey, data collection and analysis were carried out concurrently. To discover new concepts and categories, data were evaluated immediately after the in-depth interview. After carefully listening to the tape recorder to grasp each respondent's concepts, the audio recording was transcribed verbatim in Amharic and translated into English. The translated data were imported to qualitative data analysis software ATLAS ti. version 9.1 for coding. Data were analyzed using the deductive thematic analysis principle. To aid analysis, written notes and memos were linked. A total of 20 codes were grouped based on their resemblance, then themes and sub-themes were created, and a sample quotation was picked to report on.

### Operational and term definition

#### Quality of care

Quality of care is the degree to which health services for individuals and populations increase the likelihood of desired health outcomes (32).

#### Perceived quality of care for SAM management

It refers to parents' views about the functional quality of services delivered by the selected public hospital, for those admitted with SAM. It was measured by a total of 32 items of 5-point Likert scale questions. The questionnaire measured five dimensions of quality: tangibility (5 items), empathy (7 items), efficiency (6 items), safety (6 items), and improving service delivery (8 items). Respondents were allowed to score 1–5 for each item (1 = poor, 2 = fair, 3 = good, 4 = very good, and 5 = excellent). The responses of each item were summed up to get the total scores for overall and each subscale. The overall and subscale summed scores were, then, categorized into good and poor levels by using cutoff values calculated by a demarcation threshold formula.

The overall and subscale summed scores were then categorized into good and poor levels by using cutoff values calculated by a demarcation threshold formula (6, 33, 34).


$$\begin{array}{l} \text{Cut-off Value = }\frac{\text{Total highest score}-\text{Total lowest score}}{2}\\ \text{ + Total lowest score} \end{array}$$

#### Good quality

The overall level of quality was determined by computing the total from the 32 items for each respondent and using the above demarcation threshold formula as a cutoff value (threshold value) to classify the total score as good.

#### Poor quality

The overall level of quality below the demarcation threshold formula as a cutoff value (threshold value) to classify the total score as poor quality (6).

#### Caregiver

Caregiver refers to any parents, guardians, or attendants who accompanied the pediatric patient as a primary caregiver at the time of the interview.

#### Healthcare providers

Healthcare providers refer to the one who delivers care and services to the sick and ailing either directly as doctors or nurses who have contact with the management of SAM at inpatient units of pediatrics ward (35).

### Ethical considerations

Ethics approval for this study was obtained from the Addis Ababa University, College of Health Sciences, School of Nursing and Midwifery Research Ethics Committee, and the Addis Ababa City Administration Health Bureau. Written permission to carry out the research was submitted to all selected public hospitals. The objectives of the study, the risks, and the benefits of participation were communicated prior to asking about the willingness of study participants to participate in the study. Informed verbal and written consent was obtained from each respondent prior to data collection. After getting informed consent from the respondents, the right of the respondents to refuse to answer a few or all the questions was respected.

Participants were informed that their participation will be purely voluntary and were assured of the confidentiality of all information. Different measures were taken to assure the confidentiality of the study subject's response. Names or any identification were not collected. Except for the principal investigator and the research team, no other person has access to the collected data.

## Results

### Findings of the quantitative survey

#### Socio-demographic characteristics

In this study, though we expected 413 caregivers, the number of SAM cases was fewer than the calculated sample size, and a total of 181 caregivers (parents) who were found in the study period were interviewed. Out of the total participants, 134 (74.03%) were female respondents. The participants had a mean age of 30.80 ± 5.72 years. A significant number of respondents 54 (37.76%) had no formal education or secondary school. Nearly half (44.75%) of the respondents were housewives, while a minority of the respondents were private employees, accounting for 12.15% of the total participants. One hundred and twenty-one respondents (62.43% %) were from rural areas (Table 1).

**Table 1:** Socio-demographic characteristics of respondents of a study on perceived quality of care for SAM management among caregivers of under-five children in selected public hospitals in Addis Ababa, Ethiopia, 2022 (n = 181).

**Characteristics**		**Frequency (*n*)**	**Percentages (%)**
Gender	Female	134	74.03
	Male	47	25.97
Age (in years)	Mean ± SD	30.80 ± 5.72	
Relationship to child	Mother	144	79.56
	Father	26	14.36
	Other^*^	11	6.08
Educational status	No formal education	54	29.83
	Primary school	47	25.97
	Secondary school	54	29.83
	College and above	26	14.36
Marital status	Single	16	8.84
	Married	165	91.16
Religion	Orthodox	75	41.44
	Muslim	74	40.88
	Protestant	32	17.68
Place of residence	Urban	68	37.57
	Rural	113	62.43
	Housewife	81	44.75
	Farmer	42	23.2
	Government employee	36	19.89
	Private employee	22	12.15

^*^Used to indicate other relationships of attendants of the children (include brother/sister, grandmother/grandfather).

Out of the 181 children admitted with SAM, the majority were female (77.35%), newly admitted (60.22%), and aged <12 months (51.38%) with a mean age of 14.59 months (Table 2).

**Table 2:** Socio-demographic characteristics of under-five children admitted with SAM in selected public hospitals in Addis Ababa, Ethiopia, 2022 (n = 181).

**Characteristics**		**Frequency**	**Percentages (%)**
Gender	Male	140	77.35
	Female	41	22.65
Age in month (Mean ±SD = 14.59 ± 6.88)	<12	93	51.38
	12–24	88	48.62
Hospital admission	New	80	44.20
	Re-admission	101	55.80

### Perceived quality of care for SAM management

The mean Likert scale score of overall items was 3.44 with a standard deviation of ±0.27.

#### Empathy

The mean Likert scale score for this subscale was 3.36 ± 0.24. This was a lower score than the total mean (3.44 ± 0.27). The respondent rating hospital knows what he and his child want, With a mean score of 3.64 ± 0.79. The overall mean score for understanding and considering his child's condition was 3.03 ± 0.39.

#### Tangibility

For this subscale, the mean Likert scale score was 3.44 ± 0.52. The items under this dimension that were rated by respondents as poor parental perceptions availability of advanced medical equipment that needs for their child's treatment showed negative attributes (3.30). The items under this dimension that were rated by respondents high above the overall mean include physical appeal to the hospital for the treatment of the child and cleanliness of healthcare provider uniforms, and overall cleanliness of the hospital environment showed positive attributes of 3.49 ± 0.86, 3.55 ± 0.79, and 3.53 ± 0.78, respectively.

#### Safety

The mean Likert scale score for this dimension was calculated to be 3.64 ± 0.58, which was above the overall mean of the dimension. Specifically, for item statements, the highest mean score was observed for doctors' competency of not making a misdiagnosis followed by a comfortable and safe environment for patients, with 3.98 ± 1.40 and 3.90 ± 1.28, respectively. Item scales “hospital environment that is safe from infection” were rated as poor by parents, with a mean score of 3.19 ± 1.16.

#### Efficiency

The mean Likert scale score for this dimension was calculated to be 3.29 ± 0.47 which was below the overall mean of the dimension. Specifically, for the items statement, the highest treatment procedures of convenience for one's child and the lowest for reasonable medical expenses while in the hospital are 3.5 ± 0.85 and 2.64 ± 0.57, respectively.

### Degree of improvements in care services

The mean Likert scale score for this dimension was calculated to be 3.44 ± 0.37 which is almost with an overall mean score of 3.44 ± 0.27. Of the items, the highest were “the efforts for the best treatment provided by the medical staff to your child” and “health care provider efforts and willingness to prevent disease” with 3.62 ± 0.58 and 3.10 ± 1.24, respectively.

### Overall level of perceived quality of care for SAM management

Each respondent's total score from the 32 questions was calculated, and the demarcation threshold formula was used to determine whether the total score was of good or low quality. As a result, 101 (55.80%) respondents (95% CI: 48.50–63.10%) ranked the perceived quality of care for SAM management as good quality.

The perceived quality was also calculated for each quality dimension based on their subscale totals and respective cut-point values. The lowest and the highest levels of quality were for empathy and safety dimensions having 25.41 and 70.17%, respectively (Table 3).

**Table 3:** Subscale and overall level of perceived quality of care for SAM management among caregivers of under-five children at selected public hospital in Addis Ababa, Ethiopia, 2022 (n = 181).

**Component**	**No. of item**	**Mean Likert scale**	**Total score range**	**Total mean score**	**Cut-off point**	**Quality level**, ***n*** **(%)**	
						**Good quality**	**Poor quality**
Empathy	7	3.36	18–29	23.52	23.5	46 (25.41)	135 (74.59)
Tangibility	5	3.44	5–20	17.22	12.5	104 (57.46)	77 (42.54)
Safety	6	3.64	1–27	21.82	19.5	127 (70.17)	54 (29.83)
Efficiency	6	3.29	6–23	19.77	14.5	87 (48.07)	94 (51.93)
Improvements of care service	8	3.44	17–32	27.54	24.5	111 (61.33)	70 (38.67)
Overall perceived quality	32	3.44	32–160	109.87	94.5	101 (55.80)	80 (44.20)

### Factors associated with the perceived lower level of quality of care for SAM management

The logistic regression model was used to identify statistically significant independent predictors of the perceived quality of care SAM management among under-five children. Residence, hospital admission, occupation status, length of hospital stays, and categories of hospital admission were the variables that the bivariate analysis substantially associated with dependent variables. The variables that remained in the final multivariable model include age, occupation, residence, and education status of caregivers, length of hospital stay, and types of admission. However, the age of caregivers, religion, relationship of caregivers with the child, and marital status were not found in the model since they were skewed to one group.

The finding of this study showed that the odds of rating the amount of perceived low-quality care for SAM management among government employees were 2.72 times greater than participants in other occupations (AOR = 2.72, 95% CI: 1.05–7.05). As well, the odds of scoring perceived lower-quality SAM management care were 68% less likely among urban residents compared to rural residents (AOR = 0.32, 95% CI: 0.16–0.66). The odds of perceived level low quality of care for SAM management for participants that have college and higher level education were 4.42 times higher than other educational statuses (AOR = 4 .42, 95% CI: 1.41–13.86). In comparison to children who were readmitted, caregivers were 53% less likely to have a child who had a new admission rating of perceived low level of quality care for SAM management (AOR = 0.47, 95% CI: 0.23–0.94). The odds of perceived low quality of care for SAM management among those who stayed more than 7 days were 2.1 times higher than those stay <7 days (AOR: 2.1, 95% CI: 1.01–4.27) (Table 4).

**Table 4:** Factors associated with participant lower perception of quality care of SAM management among selected public hospital in Addis Ababa, May 2022.

**Characteristics**		**Perceived quality**		**OR (95% CI)**		***P*-value**
		**Poor**	**Good**	**COR (95% CI)**	**AOR (95%CI)**	
Place of residence	Rural	41	72	1	1	
	Urban	39	29	0.42 (0.23–0.78)	0.32 (0.16–0.65)	0.002^*^
Education	No formal education	33	21	1	1	
	Primary	19	28	2.3 (1.04–5.15)	1.85 (0.74–4.62)	0.187
	Secondary	21	33	2.47 (1.14–5.35)	2.03 (0.78–5.26)	0.144
	College and above	7	19	4.27 (1.53–11.90)	4.42 (1.41–13.89)	0.011^*^
Occupation	Housewife	41	40	1	1	
	Farmer	22	20	0.93 (0.44–1.96)	0.93 (0.40–2.12)	0.856
	Private employee	8	14	1.79 (0.68–4.74)	1.80 (0.61–5.28)	0.286
	Gov't employee	9	27	3.1 (1.29–7.35)	2.72 (1.05–7.05)	0.039^*^
Type of admission	New admission	41	39	1	1	
	Readmission	68	33	0.0.51 (0.28–0.93)	0.47 (0.23–0.95)	0.036^*^
Hospital stays	≤7 days	33	47	1	1	
	>7 days	22	79	2.52 (1.32–4.83)	2.1 (1.01–4.27)	0.046^*^

^*^P-value <0.05, CI, Confidence interval; COR, Crude Odds ratio; AOR, Adjusted Odds Ratio.

## Findings of the qualitative survey

### Participant characteristics

A total of 15 healthcare providers were recruited based on the saturation of ideas. The age of the participant ranged between 25 and 35 years. Regarding sex, six of them were male subjects and the rest eight were female subjects. Thirteen of them were married and their educational status revealed that 10 were BSc nurses, four were Master of Public Health (MPH) in Nutrition with a BSc nurse background, one was a general practitioner (GP), and their years of experience ranged from 2 to 10 years.

### Emerged themes

After coding in-depth interview results, the responses of study participants were categorized into four main themes and 12 sub-themes under the corresponding main themes (Table 5).

**Table 5:** Overview of theme and sub-theme in in-depth interview of study participants.

**Themes**	**Sub-themes**	**Cods**
Management process	1. Opportunities for management of SAM 2. Availability of materials and guidelines 3. Human power for the management of SAM 4. Opportunity of the health care providers	• Adequacy of human power • Availability guidelines and Equipment • Adequacy Human power • Good management for SAM • Opportunity for the child and for caregiver • Opportunity for health care providers
Factor affecting quality of care management of SAM	1. Support and attention from higher level institution 2. Supplement insufficiency 3. Lack training for health care providers 4. Lack diagnostic materials 5. Methods availing the required equipment	• Support and Attention given from higher level organization, • Lack of the training • Limited number of Health care provider • Supplement problem • Not separated room the management
Outcome of management	1. Care givers satisfaction 2. Complaints of the care givers during the management of the SAM 3. Way of solving compliant that raised from the caregivers	• Care givers satisfaction • Compliant rose during the management • Compliant solving mechanism
Quality improvement strategies	1. Way of improving quality care 2. Quality mechanism in hospital	• Quality center for monitoring • Way improving the quality care

### Theme 1: management process

Under this theme, four codes were identified. These are opportunities for management, availability of materials and guidelines, human power participating in the management of SAM, and opportunities for healthcare providers.

The management process ranged from screening to admitting the patient and depended on the criteria. A 35-year-old nutritionist stated:

“*From the beginning of malnutrition in children, we saw malnourished children come to the emergency unit and OPD. We start by measuring the anthropometrics and screening them for their nutrition status. During the screening, if they have malnutrition, especially if they have SAM and MAM, we give attention to them and admit them to the ward, especially for the SAM and complications. After the admission, we put them on milk F75 and F100.”*

“*Then, they are in the stabilization phase, and we are doing it in this way. Nutritional health education and counseling are provided for MAM. We advise them to change the nutrition status of the children so they can use the food that is found in their homes. Others, those who do not have SAM without complications, are sent to the health center for the OPT program.”*

“*This could be for the MAM. For the MAM plump let supplements and for the SAM without complications, RTUF supplements, by creating the link with the health center and treating us through the OPT program, we decided this on their nutrition status.”*

“*We admit them as inpatients or wards and treat them with F75 and F100 local, affectionately referred to as Milk. So, put them on that milk and then stabilize them until they improve their status. Then we progress from one phase to the next, first stabilizing them, then transitioning them to phase two, or rehabilitation. After their improvement, we send them again to a health center for follow-up.”* (HCP, 10)

#### Sub-theme 1: opportunities for the management of SAM

Children with SAM have received some free treatments such as therapeutic feeding and treatment for comorbidities, such as infections, pneumonia, and others. The hospital offered free care; this is an opportunity the hospital delivered. Until they were discharged from the hospital, their caregivers were fed by the hospital for free. A 29-year-old female nutritionist stated:

“*The opportunity they get here is that, like the patient mentioned their economic problem, while they stay here in our hospital, they worry about the child in the home. Some of them arise from the problem that is related to money. The service is provided for free because we consider the patient's benefits in our hospital.”* (HCP, 6)

The primary cause of malnutrition was their economic problem followed by comorbidities. They cannot afford the payment requested by the hospital for the services. A 32-year-old female BSc nurse stated:

“*The opportunity that the patient and the caregiver get from this hospital is like free treatment, including some laboratory and diagnostic tests, drugs which are found in the hospital pharmacy, therapeutic food (F75 and F100), and food and bed service for the caregiver.”* (HCP, 8)

Another 29-year-old nutritionist stated:

“*Those admitted as SAM get all services for free without any charge for bed and drugs. Additionally, the child gets a separate room from other medical cases for the purpose of decreasing the psychosocial impact for both family and child admitted.”(HCP, 7)*

#### Sub-theme 2: availability of materials and guidelines

To provide services, the hospital must have the supplies required for SAM management. The following items were required for SAM management: MUAC, tape meter, weight scale, child's monitoring chart, casserole, and cup for measuring milk, guidelines, and bucket. This study showed that these equipment are not available as per the standard.

A 32-year-old female BSc nurse stated:

“*The equipment does not exist in our hospital for example weight scale. It is difficult to measure on it. For some patients, the measured values do not exist on the board of the weight scale. For some, they do exist as we measured on it. This one is the problem. The other one is the milk measurement cup. The milk ordered per kg since we do not have the standardized cup measuring the milk. We are measuring by syringe and bottle feeding. We have MUAC.”*(HCP, 2)

A 25-year-old female BSc nurse stated:

“*For children*<*6 months old, we measure their length since they are not able to stand. The equipment for measuring the length is difficult to find in our hospital. I haven't measured yet, even though I have served 3 years here. For those able to stand, we measured their height. For those* <*6 months, we didn't measure their length because we didn't have the equipment.”(HCP, 3)*

The guidelines are required the management of SAM. A 32-year-old male BSc nurse stated:

“*There are no adequate guidelines here for the management of SAM; even the existing ones are not updated. “We didn't get an update on the management of the SAM. We sometimes use it by downloading it from the internet. We just created the protocols from what we read and, as you see, they're posted on the wall.”*(HCP, 11)

A 32-year-old female BSc nurse stated:

“*Guidelines should be posted on the wall. For example, if the physician makes an error, we will correct them by using the guidelines. Before that, we would correct them or guide them when they make an error. Whenever we see error, we just expect everything from them. We are giving care what we had trained before. If the guidelines are updated and working together, it's better.” (HCP, 4)*

#### Sub-theme 3: human power for the management of SAM

All the participants stated that there was enough human power in their organization to manage SAM. A 25-year-old female BSc nurse stated:

“*Yes, there is sufficient trained human power, but normal we should have to be updated by training. Since we are human beings, it's better if the responsible body updates us. I trained before 2 years, and they told me as trained staff and assigned us to the SAM unit and we are preparing the milk for children. Sometimes form filling is updated through time, so we just fill out the form by reading it aloud, even if they don't give us the orientation.”*(HCP3)

#### Sub-theme 4: opportunity of the healthcare providers

Almost all participants stated that there were no opportunities for healthcare providers. A 32-year-old female nutritionist stated:

“*No opportunity. Even some of us have a Master's level in nutrition; the scaled salary is not corrected at the level of the masters.”*(HCP, 8)

Another 35-year-old male nutritionist stated:

“*In relation to human power, there is no opportunity. It's hard to say there is an opportunity for healthcare providers because, there is little attention to the opportunity in shortly.”* (HCP, 10)

### Theme 2: factors affecting the quality of care for the management of SAM

Five sub-themes were discovered under this theme: lack of higher-level institution support and attention, supplement insufficiency, lack training for healthcare providers, lack diagnostic materials, and methods availing the required equipment.

#### Sub-theme 1: lack of higher-level institution support and attention to SAM management

The hospital's support from higher-level institutions (AAHB and MOH) as 32-year-old male general practitioners (MED) stated:

“*It is good but not enough. They gave training twice at different times. Even though this is good, it is not enough. In addition, all professionals should get training, and they should provide quality diagnostic materials like weight scales that are related to SAM. As it directly affects the quality of treatment, it is better if they should work on it.”* (HCP, 15)

Attention should be given to the management of SAM. A 39-year-old male BSc nurse stated:

“*it's better if a higher organization body comes and assesses us regularly. If the orientation is given to the staff, and we discuss with them and identify the gap, we can forward the solution*.” (HCP, 15)

Another 35-year-old male nutritionist stated:

“*It is hard to say its adequate. We get low support from them. And it's better if the country has its own production rather than being dependent on aid. If this gap is solved, and if F75, F100, and plumpy nut production are available it would contribute for the quality of care and problem solving.” (HCP, 10)*

Lack of attention and support from higher levels of organization to SAM management service affects the quality of care, as stated by a 35-year-old male nutritionist:

“*In my opinion, the main challenges that affect the quality-of-service provision are: first, no attention from the government from the top higher government officials to the bottom. The problem starts from that; second, there is no implementation of the food policy strategies in every organization. So, the policy should be implemented. There should not be a limit on the number of rooms if the unit is separately established as the center. If the unit is separately established, it will have its own room, supply, logistical setup, and human power.”* (HCP, 10)

#### Sub-theme 2: lack training for healthcare providers

A 29-year-old male BSc nurse stated:

“*First, the physicians are not trained, especially on the updated protocol for management of SAM. They do not follow the updated protocol even on the admission criteria for SAM. They admit the patient without fulfilling the admission criteria as SAM for services. Secondly, the factors that affect the quality are: like food dividers (milk), like preparation places; recently, in our hospital, the water supply for milk preparation is damaged, so that damaged water is not repaired as well as before, we don't get water on time, which may have a minor impact on the quality of care. So, we try to fetch water by bucket from other places, but this one is challenging for our services. Other things that affect quality care as I told additional you earlier supply problem.”* (HCP, 1)

Another 29-year-old female BSc nurse stated:

“*The factors affecting the quality of care for SAM management are the lack of trained human power and insufficiency of the equipment*.” (HCP, 13)

#### Sub-theme 3: lack diagnostic materials

Another 32-year-old male general practitioner (MD) stated:

“*The first one is the lack of quality diagnostic materials. The second one is a lack of training for SAM management. The quality of service will suffer if there are insufficient trained personnel and diagnostic materials. These are two of the major problems.”* (HCP, 15)

Problems such as a lack of laboratory investigation in the hospital where they were admitted and a lack of funds to pay for the laboratory are affecting the quality of SAM management as a 32-year-old male BSc nurse stated:

“*They can't afford the investigation. A lot of children suffering from SAM can't afford the payment required for the laboratory. They may have other comorbidities, such as cardiac cases, and when they come here, they get service for SAM for free here in our hospital, but for the cardiac service, sometimes the service is interrupted due to these problems, and this makes the long hospital stay.” (*HCP, 4)

#### Sub-theme 4: supplement insufficiency

The supplement that is needed for SAM management was insufficient according to a 32-year-old male general practitioner:

“*There is scarcity of supplements Sometimes F100 may not available and there is also a time when F 75 not available. At that time, we couldn't transfer those who have edema to transfusion. If there is no F75 we use the available thing until F75 available. The main problem is the absence of F75, F100, and plump nut which has effect on the treatment process*.” (HCP, 15)

Another 27-year-old female BSc nurse stated:

“*The factor affecting quality of care for SAM management, supplementary milk supply is a great challenge here in our hospital. For example, F100 is not adequate, this makes the treatment worse; those* <*3 months should take F100, but due to insufficiency, we're treating them with F75. So, they are not treated properly.”* (HCP, 5)

A 29-year-old male BSc nurse stated:

“*The great challenge in our hospital is the insufficiency of the equipment and, secondly, the economic status of the patient regarding the affordability of the hospital expenses. The laboratory tests are done in the hospital only for complete blood count. Other tests, like organ function tests, are done outside the hospital in a private facility. We occasionally contribute to laboratory tests because they cannot afford them. As much as possible, the hospital also supports them through the social worker.”* (HC, P9)

#### Sub-theme 5: way of availing required equipment

As a 25-year-old female BSc nurse stated:

“*The hospital should fulfill the equipment that is needed for SAM management if it doesn't have the necessary equipment's, we can't say that the patients are being treated the right way. We should measure their weight every day to assess the treatment progress. And to measure their weight, the equipment is needed.”* (HCP, 3)

Another 29-year-old female BSc nurse stated:

“*Problems of sufficient supply of milk exists in our hospital. By collaborating with the AAHB and other NGOs, supplements should be fulfilled as demand. If the supplement is sufficient, the children will be treated well, and the outcome will be better*.” (HCP, 5)

As 29-year-old female nutritionist stated:

“*To avail the needed materials, the concerned bodies (government, community, and the staff) should see a way of solving this problem. For the government hospitals, to make out resource systems likes other departments, for instance, laundry, and other clinics, it's better if service is given based on the affordability of the client. Since those laboratory tests are expensive in private hospitals, outsourcing is needed for the laboratory test service with the payment that most can afford. For example, if Zewidtu Memorial Hospital outsources the patient's test, the patient will not suffer the payment needed for the test. For example, the Wudassie diagnostic center is outsourced to the Zewidtu memorial hospital. First, the expense is low, and the patient uses that opportunity. The government should make a plan for outsourcing the needed materials. The laboratory should be outsourced. The quality would be enhanced, there would be competition with suppliers, the government would minimize human resources and many patients would have benefited.”* (HCP, 7)

### Theme 3: outcome of quality of care for SAM management

Under this theme, their sub-themes were discovered. These are caregiver satisfaction, complaints of the caregivers during the management of the SAM, and ways of solving the complaints that arise from the caregivers.

#### Sub-theme 1: caregivers satisfaction

Almost all the participants stated that the caregivers were satisfied with the care they give to their children. A 35-year-old male nutritionist stated:

“*The satisfaction is great, and they are happy with the support that we have at hand which are health education and counseling on the food preparation and on the treatment they received. They are happy. They get the service for 8 or 15 days, depending on the improvement of the child. Since the children improved during those days of treatment, they are very happy and satisfied.”* (HCP, 10)

#### Sub-theme 2: complaints of the caregivers during the management of the SAM

According to the healthcare providers, the complaints that arise by the caregivers are due to prolonged hospital stay, lack money to afford the required payment, and worry about the family they left at the home. A 32-year-old female BSc nurse stated:

“*The complaints are that some of them come from far away, and some of the treatments take longer time and they haven't sufficient money, so they worry for family left at home. They complain for longer hospital stay, and they want to go home.”* (HCP, 2)

A 29-year-old female nutritionist stated:

“*Compliant rose in the process of treatment are: discharge earlier; worry about the family left at home; economic etc.” (HCP, 7)*

#### Sub-theme 3: way of solving complaints raised by caregivers

The complaints raised by the caretakers will be resolved with the help of the administrator and social workers. A 37-year-old male nutritionist stated:

“*First, the problem will be identified, by a unit leader or group leader and will be tried to slove [SIC] the problem. If the complaint is not solved in the unit and it needs budgets, it will be submitted to the concerned body. It will be presented to the pediatrics directorate and if necessary to higher officials according the request.”* (HCP, 10)

A 32-year-old male BSc nurse stated:

“*We try to manage their complaints here in the ward. For instance, if the complaint was about money for transportation, we mobilizing the staff. We make solutions for problems that we can solve. But if not, we connect them to the social worker, and they connect with hospital management.”* (HCP, 11)

### Theme 4: quality improvement strategies

Two codes are identified under this theme. These are ways of improving the quality of care for SAM management and quality mechanism in the hospital.

#### Sub-theme 1: way of improving quality of care for SAM management

The proposed ideas to enhance the quality of care for SAM management include fulfilling the required materials, training the healthcare providers, teaching the community about malnutrition and prevention methods, and supporting families of children with SAM if the cause of the malnutrition is economic. A 32-year-old female BSc nurse stated:

“*To enhance the quality of care of SAM management, first training must be given for health care providers, secondly room of the patients must be kept clean, thirdly the materials needed for the proper management should be fulfilled the other thing is getting all investigation and the drugs here in our compound.”* (HCP, 4)

A 32-year-old male general practitioner (MD) stated:

“*To improve the quality of care provided by SAM management, every personnel working in the pediatric ward should receive training. Second, the number of employees should be greatly raised. Third, as I already stated, proper class size is required. And, sufficient diagnostic materials are also needed. Collaboration with health centers could also be an option. To spot the problem early, meet with health facilities on a regular basis and do community assessment surveillance. For example, treating people on the border as MAM before they acquire SAM improves national quality.”* (HCP, 15)

A 29-year-old female nutritionist stated:

“*In their homes, the children should be supported. Before they develop the difficulty, the government must take action at the national level. Community-wide prevention efforts should be bolstered. Preventative measures should include ANC, pregnancy, and immunization. This is where I believe the divide exists. Breastfeeding and supplementary feeding must be taught up to the age of 2 years*.” (HCP, 7)

The media should give nutrition awareness in the community, and the nutrition department should own media (channel) at the country level. A 37-year-old male nutritionist stated:

“*Media should give nutrition awareness in the community and the nutrition department should own media (channel) as country level. Nutritional education should be given to the community through media. If the food production is established in the country level, like the production therapeutic feeding, then the accessibility of the therapeutic feeding will increase to where it is needed, and quality will be improved. Then the problem with malnutrition will be solved. Additional, policy implementation, which is related to the nutrition should be done, and government should give attention to things to be done in the community level especially on the prevention, and separate unit should be established.”* (HCP, 10) [SIC]

#### Sub-theme 2: quality assurance mechanism in hospital

All participants stated that they have a quality center in their hospital. The assessments were done using standard checklists, inquiries with caregivers, and data registration will be checked for fullness. A 29-year-old male nutritionist stated:

“*They come to the ward and assess us through their checklist. And ask the patient how they the care was given to them*.” (HCP, 9)

A 32-year-old male BSc nurse stated:

“*For quality assessment, the mechanism that they use in our hospital is, morning session. We discuss this with our staff and data registration will be checked for the fullness. This is how quality assessment is done in our hospital.”* (HCP, 11)

## Discussion

This study investigated the level of perceived quality of care for SAM management and possible determinant factors among caregivers of under-five children. The results showed that caregivers perceived the quality of care for SAM management to be low. The logistic regression analysis showed that low perceived quality of care of SAM management was significantly associated with being an urban resident, having a higher education level, being a government employee, readmission to the hospital, and longer hospital stays. To the best of our knowledge, studies that assessed the perceived quality of care for SAM management are rare, and this study provides an important contribution to the current literature.

In this study, above half of the interviewed caregivers (55.80%) perceive that SAM management is of good quality. Similar findings were reported in previous studies which were conducted at different hospitals in Ethiopia and Kenya (6, 34, 36). However, compared to findings from India, 88.9% of the study participants thought the service was of high quality (37). This discrepancy may be due to the different study environments, India has a more developed healthcare system than Ethiopia, as well as the fact that the earlier study only reported on the general level of healthcare service quality, whereas the current study concentrated specifically on SAM management. The mean score of overall perceived quality (3.44 ± 0.27) in this study was generally lower than the national plan (38). Additionally, the score was also lower than that of Israel, which reported an overall mean parental perception score of 4.35 ±0.43 (39). The variation in organizational structure and underlying economic concerns of hospitals could be the cause of this disparity.

Regarding tangibility as a quality dimension, this study found an overall mean score of 3.44 ± 0.52 which is equal to the overall mean (3.44). This indicates that physical quality is improving, though it was lower than findings from studies in Bangladesh (3.49 ± 1.01) and Vietnam (3.95 ± 0.60) (6, 40, 41). This finding was also higher than that of a study in southern Ethiopia (2.77 ± 0.66) (6). The possible reason for this disparity could be differences in infrastructure and facilities across the hospitals being evaluated in which better infrastructure is likely to be available in Addis Ababa, which is the capital city of the country.

This study demonstrated that the availability of advanced medical equipment that is needed for childcare has negative valence (mean = 3.28 ± 0.97), and healthcare providers indicated the scarcity of equipment needed for SAM management. This finding was supported by studies conducted in Kenya, India, and Burkina Faso which reported the availability of advanced medical equipment as one of the most significant barriers to the delivery of high-quality healthcare services (37, 40, 42). Furthermore, empathy and safety factors have the lowest (25.41%) and highest (70.17%) levels of quality out of all the dimensions. This was lower than a study conducted in Vietnam (41). This disparity might be due to the difference in the management system of the hospital and working environment which implies that the importance of people-centeredness in the healthcare system was not adequately emphasized and addressed. It is a far cry from the theoretical framework and health-system strategy that states that healthcare facilities must strive for high performance by focusing on the quality of their services (32).

This study finds that parents with a higher educational background were more likely to judge the quality of healthcare services as poor. This finding was in line with previous studies conducted in Ethiopia (34, 43) and other countries (36, 39, 44). The gap in their perception and expectation of the healthcare services that they are really receiving could be the explanation for these findings. Additionally, similar to a study conducted in Germany (45), government-employed caregivers were also more likely to rate the quality of healthcare as poor. This similarity could also arise from the fact that government employees are more likely to be educated and have better media access on quality and a better sense of quality problems. This was also supported by the qualitative study findings in which healthcare providers also admitted that the provision of quality care for the SAM management was not possible due to the lack of support and attention from higher levels of institutions, and lack of supplements, separate unit, laboratory facilities, and training, shortage of staffs. This finding was supported by earlier studies in Northwest Ethiopia, Bangladesh, Iran, Malawi, Ghana, and South Africa (3, 14, 40, 46–50). However, unlike the findings of the earlier study in Northwest Ethiopia (6), which reported that urban residents were more likely to perceive the service quality as poor, this study found that urban residents were less likely to rate the service as low quality. The possible reason for this disparity could be a language barrier in which rural residents in the present study were from the Oromia region, while the previous study included participants from the same region.

In this study, parents who stayed longer than 7 days were more likely to rate the quality of healthcare as poor or inefficient than others. This finding was supported by earlier studies conducted in Ethiopia and Michigan (51, 52). This might be because those who stayed longer might have higher exposure to mistreatment and financial burden which could affect their satisfaction. The qualitative findings also identified that caregivers' complaints stem from a protracted hospital stays. Moreover, parents who were readmitted to the hospital were more likely to give the quality of care a higher rating than those who were hospitalized for the first time. A similar finding was reported in a study conducted in China (53). This could be because of the fact that newly admitted caregivers may not find the service they expected, while those with previous experience might shape their expectations as per their prior exposure. This study was not free of limitations, and the results should be interpreted cautiously. The smaller sample size might have affected the power in this study, and caregivers might not be able to measure the competencies of healthcare provider based on this model, and the study aimed on functional aspects of healthcare services delivery and technical parameters were not well-addressed. In addition to this, employment of the mixed approach matrix was the study's strength, where we collected data from both healthcare provider and caregivers, which increases the data validity.

## Conclusion

In this study, the perceived quality of SAM management services was low against the national goal of quality improvement to meet the expectations of both internal and external clients. The quality of SAM management services was challenged by the lack of support and attention from higher levels of institutions, lack of supplements, lack of separate units, lack of laboratory facilities, and shortage of training for healthcare providers. Rural residents, those with more educational qualifications, government employees, newly admitted patients, and patients who stayed longer in hospitals were the most unsatisfied groups. Hence, improving support and logistic supply to health facilities, providing client-centered care, and responding to caregivers' demands are recommended to improve quality and satisfaction.

## Data availability statement

The raw data supporting the conclusions of this article will be made available by the authors, without undue reservation.

## Ethics statement

The studies involving human participants were reviewed and approved by Addis Ababa University, College of Health Sciences, and School of Nursing and Midwifery research Ethics Committee and Addis Ababa City Administration Health Bureau. Written informed consent to participate in this study was provided by the participants' legal guardian/next of kin. Written informed consent was obtained from the individual(s), and minor(s)' legal guardian/next of kin, for the publication of any potentially identifiable images or data included in this article.

## Author contributions

BA, NA, and FA: study conception and design, analysis, and interpretation of results. BA: data collection. NA and FA: draft manuscript preparation. All authors reviewed the results and approved the final version of the manuscript.
